# Towards healthy learning climates in postgraduate medical education: exploring the role of hospital-wide education committees

**DOI:** 10.1186/s12909-017-1075-0

**Published:** 2017-12-06

**Authors:** Milou E. W. M. Silkens, Kiki M. J. M. H. Lombarts, Albert J. J. A. Scherpbier, Maas Jan Heineman, Onyebuchi A. Arah

**Affiliations:** 1 Professional Performance Research group, Department for Educational Support, Academic Medical Centre/University of Amsterdam, Meibergdreef 9 1100DE, Amsterdam, The Netherlands; 2 Department of Educational Development and Research, Maastricht University, Box, 616 6200 MD Maastricht, The Netherlands; 3 Department of Epidemiology, Fielding School of Public Health, University of California, Los Angeles (UCLA), 650 Charles E. Young Drive South, Los Angeles, CA 90095 USA; 4 UCLA Centre for Health Policy Research, 10960 Wilshire Boulevard, Los Angeles, CA 90024 USA

**Keywords:** Learning climate, Postgraduate medical education, Quality control, Quality improvement, Educational governance

## Abstract

**Background:**

Postgraduate medical education prepares residents for delivery of high quality patient care during training as well as for later practice, which makes high quality residency training programs crucial to safeguard patient care. Healthy learning climates contribute to high quality postgraduate medical education. In several countries, modernization of postgraduate medical education has resulted in hospital-wide responsibilities for monitoring learning climates. This study investigates the association between the actions undertaken by hospital-wide education committees and learning climates in postgraduate medical education.

**Methods:**

Research conducted in December 2010 invited 57 chairs of hospital-wide education committees to complete a questionnaire on their implemented level of quality improvement policies. We merged the survey data from 21 committees that oversaw training programs and used the Dutch Residency Educational Climate Test (D-RECT) instrument in 2012 to measure their training programs’ learning climate. We used descriptive statistics and linear mixed models to analyse associations between the functioning of hospital-wide education committees and corresponding learning climates.

**Results:**

In total, 812 resident evaluations for 99 training programs in 21 teaching hospitals were available for analysis. The implementation level of the internal quality management systems as adopted by the hospital-wide education committees varied from 1.6 to 2.6 on a 5 point Likert-scale (ranging from 1 (worst) to 5 (best)). No significant associations were found between the functioning of the committees and corresponding learning climates.

**Conclusions:**

The contribution of hospital-wide committees to creating healthy learning climates is yet to be demonstrated. The absence of such an association could be due to the lack of a Plan-Do-Check-Act cycle guiding the policy as implemented by the committees and the lack of involvement of departmental leadership. Insight into the impact of these strategies on learning climates will benefit the quality of postgraduate medical education and, hopefully, patient care.

## Background

High quality postgraduate medical education (PGME) is of importance to safeguard patient care as training programs prepare residents to deliver high quality patient care during their training period and thereafter [1]. An important indicator of PGME quality is the learning climate, which is known to influence a variety of residents’ outcomes, such as training satisfaction, the use of knowledge and the development of a professional identity [2–7]. The learning climate can be described by residents’ common perceptions about the atmosphere of a department [8] and training policies, practices and procedures [9]. The importance of facilitating healthy learning climates (referring to a learning climate that supports residents’ learning and development) throughout residency training has been acknowledged by accreditation councils [10, 11] (i.e. the Accreditation Council for Graduate Medical Education) and professional bodies alike [12, 13] (i.e. the Royal Dutch Medical Association). As such, several healthcare systems have implemented hospital-wide responsibilities to monitor and improve the quality of learning climates [10, 12]. However, insights in the effects of these responsibilities for the quality of PGME, and more specific for learning climates, are lacking.

For patient care, hospital-wide responsibilities for quality monitoring and improvement are named under the umbrella term ‘clinical governance’ [14, 15]. Clinical governance is aimed at integrating quality assurance and quality improvement efforts throughout a hospital in order to facilitate high quality patient care [14, 15]. Following the example of clinical governance, some healthcare systems ratified legislation requiring teachings hospitals to have a hospital-wide education committee (HEC) as a way to effectuate ‘educational governance in PGME’ [12]. The role of such committees is to monitor continuous quality assurance and improvement initiatives aimed at achieving high quality PGME, and high quality learning climates specifically [12].

Previous research into quality assurance and improvement efforts in PGME as initiated by clinical departments, showed positive results for the teaching performance [16] of medical specialists, as well as on learning climates [17]. Although it was speculated that a well-functioning HEC will contribute to such departmental quality efforts [17], there is no evidence of the intended effects of HECs. Therefore, the true value of such committees for learning climates remain unclear. In this study, we performed an initial exploration of whether the functioning of the HEC was associated with the learning climate as perceived by residents.

## Methods

### Setting

The current study focused on teaching hospitals providing residency training in the Netherlands. The Royal Dutch Medical Association is responsible for the regulation of Dutch PGME. Legislation enacted in 2011 requires teaching hospitals to have a HEC specifically responsible for monitoring and promoting the quality of PGME. Although many teaching hospitals institutionalized a HEC before 2011, regulations about the responsibilities of these committees were absent before 2011. From 2011 onwards, HECs should represent all residency program directors, residents and the hospital board [12], but may also include other supporting staff. Also, HECs have to meet a minimum of four times a year and dedicate their activities to facilitating high quality training for the collective resident group (for example by preparing internal audits of training programs) and protecting individual residents (for example by mediating conflicts between educators and residents and safeguarding residents’ interest) [12]. Furthermore, HECs monitor quality improvement initiatives for PGME as performed within the teaching hospital. A core task of HECs is to guarantee healthy learning climates within their teaching hospital.

### Participants and data collection

For this study we used and combined two separately collected datasets. One contained data that were collected as part of a study on the performance of HECs in Dutch teaching hospitals [18]. The other dataset contained outcomes of the Dutch Residency Educational Climate Test (D-RECT) questionnaire, which was used by training programs to evaluate their learning climate. We used both HEC data and D-RECT data to perform a cross-sectional analysis exploring the association between the functioning of the HEC and the learning climate perceptions of residents.

To acquire the HEC data, chairs of the HEC committees (*N* = 57) were invited between December 2010 and February 2011 to fill in a paper-based questionnaire on the functioning of the HEC. Chairs were approached by regular mail and reminded up to three times to fill in the questionnaire. The D-RECT learning climate data were collected by the residency training programs as part of their regular quality improvement initiatives. The number of training programs performing a D-RECT evaluation varied per hospital. Participating training programs invited all residents to complete the D-RECT via a web-based system and during a pre-determined period (commonly 1 month). Residents received a maximum number of three automatically generated reminders. Occasionally, training programs would invite doctors who were not in training and fellows to complete the D-RECT as well (in addition to residents). These evaluations were included in the study and thereby, we followed the program directors’ judgment about which respondents contributed. Given that the new legislation regarding HEC’s quality monitoring role in residency training came into effect January 1st 2011, we assumed it would take at least 1 year for an effect on the learning climate to become visible. Therefore, we only included the 2012 D-RECT data.

Teaching hospitals using the D-RECT to evaluate the learning climate of training programs were asked to sign informed consent by which permission was provided to use the anonymous D-RECT data for research purposes. For teaching hospitals that refused to sign informed consent the D-RECT was still available, but evaluation results would not be provided to the research group. For residents, participation in both questionnaires was voluntary and anonymous. According to Dutch law on medical research (Medical Research Involving Human Subjects Act (WMO)), individual informed consent was deemed unnecessary. The institutional ethical review board of the Academic Medical Centre of the University of Amsterdam assessed the study protocol and provided a waiver for formal ethics approval (reference number: W14_065 # 14.17.0090).

### HEC questionnaire

Lombarts et al. [18] developed a questionnaire to evaluate the policies and procedures implemented by the HEC. A detailed description of the development, content and results of the complete questionnaire is provided elsewhere [18]. For the current study, we focused on those questions that evaluated the functioning of the HEC by considering the level of implementation of the internal system of quality management and the anticipation of upcoming quality changes during meetings.

The internal system of quality management as carried out by the HEC was assessed by five questions, derived from a national report on quality indicators for PGME [19], requesting a rating of the level of quality control as performed by the committee. The questions addressed the following topics: (1) the learning climate for residents, (2) the working climate for the teaching faculty, (3) the extent to which the committee promoted internal quality control, (4) the way in which the committee handled the complaints of residents and (5) the extent to which the committee functioned as an effective team. These questions could be answered on a five-point Likert scale (range 1 to 5), where the fifth and highest level of the scale suggests a well-functioning HEC using a Plan-Do-Check-Act (PDCA) cycle to guide policies [20] and lower ratings presenting HECs displaying less systematic or developed approaches towards PGME quality management.

The activity of the HEC with anticipated quality changes (anticipation-score), referring to the upcoming new legislation, was determined by counting responses to the question asking what soon to be mandatory quality aspects were discussed by the committee during their meetings. Based on discussion within the research team, six quality aspects were selected as being most relevant for our study, namely: (1) PGME modernization, (2) writing local plans for education [12], (3) distribution of residents across teaching hospitals [21], (4) using a PDCA cycle [20], (5) using the D-RECT [22, 23] and (6) using the System of Evaluation of Teaching Qualities [24, 25]. One point was assigned to each topic addressed in the meeting. Eventually, the anticipation score could range from zero to six, with six representing a HEC that reached the highest level of anticipation.

### The D-RECT

The D-RECT is a 35-item questionnaire, covering nine learning climate domains: educational atmosphere, teamwork, role of specialty tutor, coaching and assessment, formal education, resident peer collaboration, work is adapted to residents’ competence, accessibility of supervisors, and patient sign-out [23]. The aim of the D-RECT is to identify areas that can benefit from improvement initiatives targeted at the learning climate in PGME. The items of the D-RECT can be answered on a five point Likert-scale (1 = totally disagree, 2 = disagree, 3 = neutral, 4 = agree, 5 = totally agree). Additionally, a ‘not applicable’ option is provided.

### Data analysis

We described the main characteristics of the study population using descriptive statistics and frequencies. Since a minimum of three evaluations are needed for a reliable mean total score of the D-RECT [23], training programs with less than three resident evaluations were removed from the dataset. Resident evaluations with more than 50% missing questions (>17 questions) were excluded from further analysis. Otherwise, missing values were assumed to be missing at random and imputed by using expectation-maximization (EM). An average composite score was computed for the 35 items of the D-RECT representing the overall learning climate.

To perform an initial exploration of whether the functioning of the HEC was associated with the D-RECT scores of training programs, unadjusted and adjusted linear mixed models with random-intercepts were used. For the adjusted model, the type of hospital (academic – providing top clinical care, scientific research and PGME as well as coordinating PGME for affiliated hospitals; top clinical teaching hospitals – providing top clinical care, scientific research and PGME; and general teaching hospitals – providing patient care and PGME) was taken into account as a hospital level covariate. Resident level covariates were the gender of the resident and the corresponding residency year.

Resulting associations were reported as regression coefficients (b) and their 95% confidence intervals (95% CI). We performed all analyses using SPSS Statistics version 20 (IBM Corp).

## Results

Of the 57 invited HECs, 50 (88%) participated in the HEC questionnaire. Corresponding D-RECT data were available for 21 teaching hospitals. Of all residents that were invited by these 21 hospitals, a total of 834 residents responded (response rate of 71%). After exclusions, 812 resident evaluations for 99 training programs in 21 hospitals were available for analysis. A detailed description of the study sample is provided in Table 1.

**Table 1:** Descriptive of the study population

Characteristics	Study sample
Number of evaluations, n	812
Male respondents, n (%)	348 (42.9)
Female respondents, n (%)	462 (56.9)
Missing, n (%)	2 (0.2)
Type of respondent	
Residents, n (%)	690 (84.9)
Doctor not in training, n (%)	115 (14.2)
Fellow, n (%)	7 (0.9)
Number of training programs evaluated, n	99
Number of teaching institutes, n	21
Academic, n (%)	3 (14.3)
General, n (%)	8 (38.1)
Top clinical teaching hospitals, n (%)	10 (47.6)
Resident year of training, n (%)	
1	174 (25.2)
2	146 (21.2)
3	117 (17.0)
4	105 (15.2)
5	92 (13.3)
6	56 (8.1)
Overall learning climate score, mean (SD^a^)^b^	3.8 (0.5)
Number of HECs participated, n (% of all 57 invited HECs)	50 (87.7)
Anticipation-score, mean (SD)^c^	2.7 (1.4)
Internal system of quality management^d^	
Residents’ learning climate, mean (SD)	1.7 (1.2)
Faculty’s climate, mean (SD)	1.6 (1.0)
Internal quality control, mean (SD)	2.6 (1.0)
Handling of complaints, mean (SD)	1.7 (1.0)
Effective team, mean (SD)	1.6 (0.7)

^a^ *SD* Standard deviation

^b^Score range 1 (worst) to 5 (best)

^c^Score range 0 (worst) to 6 (best)

^d^Score range 1 (worst) to 5 (best)

The overall D-RECT score in the sample was 3.8 (Table 1). The responses to the five questions evaluating the implementation level of the internal quality management system as adopted by the HEC varied from 1.6 (HEC functions as an effective team) to 2.6 (promotion of internal quality management) (Table 1). The mean anticipation-score was 2.7 (Table 1). No significant associations were found between the functioning of the HEC and corresponding learning climates (Table 2).

**Table 2:** Association between the HEC functioning and corresponding learning climate perceptions evaluated by D-RECT

	Learning climate perceptions evaluated by the D-RECT (overall scale)			
	Unadjusted		Adjusted^a^	
Functioning of the HEC	Regression coefficient b (95% CI)	*P*-value	Regression coefficient b (95% CI)	*P*-value
Residents’ learning climate	0.01 (−0.09 – 0.11)	0.84	−0.03 (−0.15 – 0.09)	0.62
Faculty’s climate	−0.05 (−0.19 – 0.07)	0.37	−0.02 (−0.17 – 0.12)	0.75
Internal quality control	0.01 (−0.09 – 0.11)	0.87	0.04 (−0.07 – 0.14)	0.51
Handling of complaints	−0.02 (−0.11 – 0.07)	0.63	−0.08 (−0.18 – 0.03)	0.15
Effective team	0.08 (−0.02 – 0.19)	0.11	0.05 (−0.09 – 0.20)	0.49
Anticipation-score	−0.04 (−0.08 – 0.01)	0.09	0.04 (−0.04 – 0.12)	0.36

^a^ Adjusted for hospital type, resident gender and resident year of training

## Discussion

Due to its explorative nature, this study should be considered a first evaluation of the value of HECs monitoring learning climates in PGME. Our study did not show a significant association between the functioning of the hospital-wide education committee and the D-RECT learning climate scores of training programs. We can think of several explanations for our reported results.

Firstly, our results show that the scores referring to the internal system of quality management as carried out by the HECs were low (<3 on a 5-point scale ranging from 1 (worst) to 5 (best)), suggesting the HECs did not use a well-developed PDCA cycle to guide their policies. These findings might explain the lack of association between the HEC and corresponding learning climates, especially since Heard et al. demonstrated the success of a cyclical system involving annual evaluation of PGME quality, execution of subsequent action plans and thorough follow up on questionnaire results regarding residents’ work environments [26]. They conclude that teaching hospitals should align organizational policies with the PDCA cycle in order to enact sustainable change [26]. Since HECs participating in the current study lacked a system of internal quality management aligned with the PDCA cycle, actual impact of the HEC on learning climates in PGME might be absent.

Secondly, research into successful quality management of patient care shows that involvement of organizational-level bodies (e.g. hospital-wide committees) alone is not enough to bring about change at the work floor [27, 28]. In the end, patient care is delivered at clinical departments and the involvement of departmental leadership is crucial to improve patient care practice [27, 28]. Similarly, learning climates are constructed at the level of the department and involvement of departmental leadership for PGME might be necessary to impact these climates. Since legislation regarding the responsibilities of the HEC came into force in 2011, it might be that cooperation and alignment between the organizational level (the HECs) and the department level (heads of departments or departmental leadership for PGME) was not yet fully developed.

Although our study does not show an association between the functioning of the HEC and the learning climate of training programs, there might be value in hospital-wide committees focusing on PGME quality nonetheless. Especially since literature shows promise for similar approaches towards assuring PGME quality [26, 29, 30], our study might have failed to capture the full impact of HEC policies. As the PGME is provided in a complex setting (the healthcare system), there are many factors besides HECs that might benefit and impair the quality of PGME. Although we succeeded in taking into account several of these factors as confounders in our analyses, our results should still be interpreted with caution.

Furthermore, we have used existing data in the current study, which might have limited the scope of our investigation into the functioning of the HECs. This is illustrated, for example, by the fact that we had to assume that when HECs were discussing anticipated quality changes (anticipation-score), they would also act upon these topics. In other words: the anticipation-score in this study was used as a proxy for the actual functioning of the committees. Moreover, the questionnaire that was used to investigate the HECs used self-perceptions of the HECs regarding their policies and practices and, as such, may be subject to measurement error or reporting bias. Even more so, since the committees were about to be assigned a legal responsibility to monitor training programs at the time of questionnaire administration, answers provided by the committees might be positively biased. Taking these limitations into account, we state that the HEC data used in this study has its limitations and, therefore, should be considered to be a first exploration of the functioning of HECs.

Moreover, the current study only represents a subset of residents that were trained at the included teaching hospitals. Based on self-reported data by the teaching hospitals in 2010, a total number of 2178 residents were trained. This means that the 812 residents that replied to the D-RECT in the current study represent approximately 40% of the total resident group. We still consider this a substantial group of residents and have no reason to expect that this subset of residents is different from the other 60%. Alternative methods for evaluating learning climates are used within the Netherlands, meaning that some training programs (and corresponding residents) will not take part in the D-RECT and are therefore not included in the current study. Still, we advise caution when extrapolating the results presented in this study to the overall resident population.

Finally, given that the new responsibilities of the HEC only became into force in 2011 and HECs were not yet actively discussing soon to be mandatory quality aspects (2.7 on a 6-point scale ranging from 0 (worst) to 6 (best)), the current exploration might be too early to actually demonstrate the effect of the HECs on corresponding learning climates. We recommend future research to focus on a longitudinal follow-up, exploring both the longitudinal development of the functioning of the HEC as well as the longitudinal development of corresponding learning climates.

## Conclusions

Since strategies like the implementation of education committees for PGME and systematic organizational-level policies (including the use of a PDCA cycle by the HEC) are increasingly used around the world, insight into the impact of such strategies is needed. Our study shows that the internal system of quality management as carried out by the HECs was premature (scores <3 on a 5-point scale ranging from 1 (worst) to 5 (best)). Furthermore, our research did not find a substantial association between the functioning of HECs and corresponding learning climates. Future research could focus on selecting appropriate indicators to measure the impact of organizational strategies, exploring the functioning of HECs longitudinally, and on providing insight into how HECs operationalize their responsibilities and affect outcomes.

## Abbreviations

D-RECT: Dutch Residency Educational Climate Test
EM: Expectation-maximization
HEC: Hospital-wide education committee
PDCA cycle: Plan-Do-Check-Act cycle
PGME: Postgraduate medical education
WMO: Medical Research Involving Human Subjects Act
